# Ectopic Papillary Thyroid Carcinoma of the Posterior Pharynx 

**DOI:** 10.22038/IJORL.2023.73099.3471

**Published:** 2023-11

**Authors:** Chang Haur Lee, Firdaus Hayati, Nornazirah Azizan, Siti Zubaidah Sharif

**Affiliations:** 1 *Breast and Endocrine Unit, Department of Surgery, Queen Elizabeth II Hospital, Ministry of Health Malaysia, Kota Kinabalu, Sabah, Malaysia.*; 2 *Department of Surgery, Faculty of Medicine and Health Sciences, Universiti Malaysia Sabah, Kota Kinabalu, Sabah, Malaysia.*; 3 *Department of Pathology and Microbiology, Faculty of Medicine and Health Sciences, Universiti Malaysia Sabah, Kota Kinabalu, Sabah, Malaysia.*

**Keywords:** Case report, Papillary thyroid carcinoma, Pharyngeal neoplasms, Thyroid neoplasms

## Abstract

**Introduction:**

Ectopic thyroid is an uncommon condition resulting from the aberrant development of the normal thyroid gland and is usually found along the thyroglossal tract: lingual, submandibular, thyroglossal cysts, intra-tracheal and mediastinal, or, on rare occasions, in the adrenal gland, gallbladder, gastrointestinal tract, pancreas, and struma ovarii.

**Case Reports:**

We describe a novel case where primary papillary thyroid carcinoma (PTC) was found after a trans-oral excision of a tumor containing ectopic thyroid tissue at the posterior pharynx, an area not known to be a location for ectopic thyroid. Delays due to the COVID-19 pandemic resulted in regional cervical metastases and multifocal PTC. The female patient successfully underwent total thyroidectomy, selective cervical and central lymph node dissection, followed by adjuvant radioactive iodine ablation, with no evidence of distant metastases.

**Conclusions:**

Ectopic thyroid tissue is uncommon and may be in the posterior pharynx. The principles of management remain those of differentiated thyroid malignancy: complete surgical resection of any tumor focus, total thyroidectomy, and node dissection of involved lymph nodes, followed by adjuvant radioactive iodine in iodine-sensitive tumors.

## Introduction

Ectopic thyroid tissue results from the aberrant development of the normal thyroid gland during embryogenesis with a prevalence of 1 per 100,000 to 300,000 (1). Most ectopic thyroid tissues are found along the thyroglossal tract (90%); lingual, submandibular, thyroglossal cysts, intratracheal, and mediastinal (1). 

Uncommon sites (10%) reported from the literature include the adrenal gland, gallbladder, gastrointestinal tract, pancreas and struma ovarii. Along with the normal thyroid gland, ectopic thyroid tissue is similarly predisposed to developing malignancy and differentiating primary malignancy from metastatic disease in these sites may sometimes be challenging. 

We describe an unusual case of papillary thyroid carcinoma (PTC) in ectopic thyroid tissue located in the posterior pharynx which has not been reported before in the literature and discuss the clinical implications of ectopic thyroid.

## Case Report

A 53-year-old lady presented to the otorhinolaryngology clinic with complaints of foreign body sensation at the back of the throat for the past 2 years, which had increasingly worsened over the past 2 weeks. She did not have any hoarseness of voice, dysphagia, or odynophagia. She was otherwise well, with no constitutional symptoms of malignancy. 

**Fig 1:**
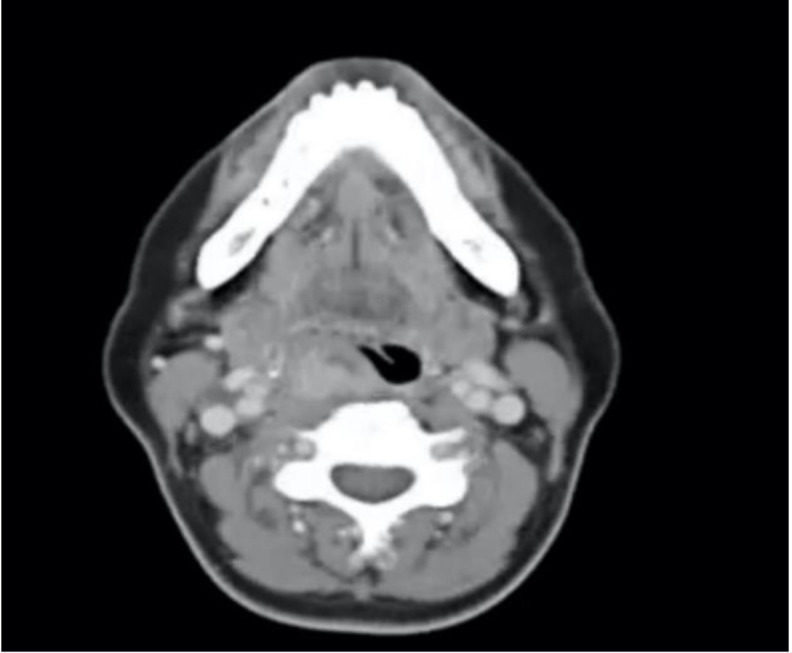
Axial CT image showing tumour at the right posterior pharynx

A clinical examination of the neck revealed neither an enlarged thyroid gland nor palpable cervical lymph node. The systemic examination was otherwise unremarkable. Indirect laryngoscopy showed a soft tissue lesion in the posterior pharynx with medialization of the right vocal cord. Routine bloodwork and thyroid hormones were normal. A contrast-enhanced computed tomography (CT) of the neck showed a right supraglottic enhancing mass within the pyriform fossa measuring 2 x 1.8 x 2.2 cm (Fig. 1). 

**Fig 2:**
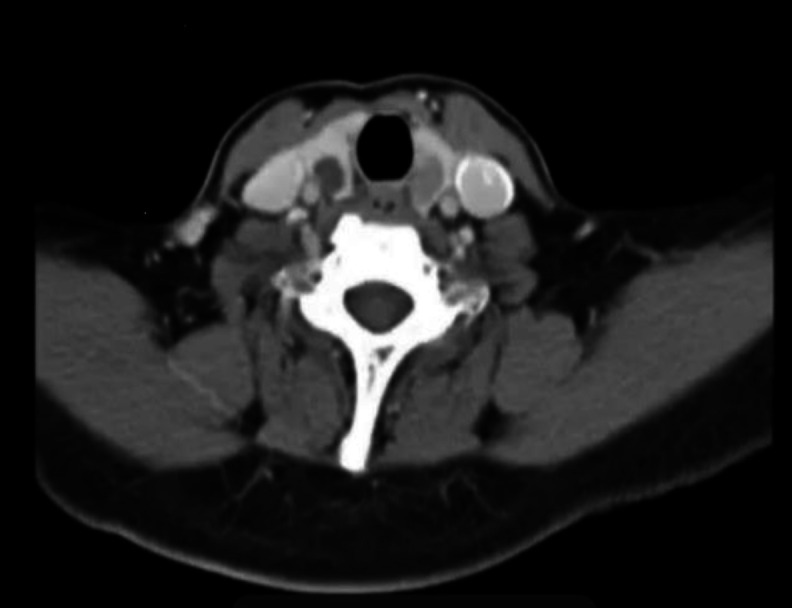
Axial CT image showing bilateral thyroid nodules

Two small thyroid nodules were found on the right (0.2 x 1.0 cm) and left (1.3 x 1.3 cm) (Fig. 2). 

Ultrasonography (USG) of the neck was done and reported benign-looking right ACR-TIRADS 1 (0.5 x 0.8 x 1.0 cm) and left ACR-TIRADS 2 (0.4 x 0.5 x 0.6 cm) nodules with normal-appearing sub-centimeter cervical lymph nodes. 

**Fig 3:**
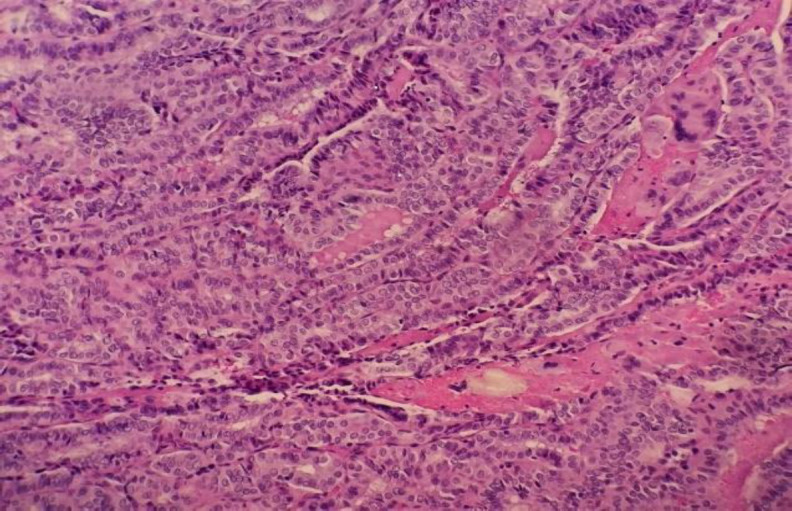
The posterior pharynx ectopic thyroid tissue in papillary structure with the presence of nuclear grooving and intranuclear inclusion representing a PTC (x20 magnification).

An initial tissue biopsy of the posterior pharyngeal mass showed benign mucosal tissue. A trans-oral excision of the posterior pharyngeal tumor was then performed, in which histopathological examination (HPE) revealed a fibrocollagenous tumor infiltrated by neoplastic cells with classical nuclear features of papillary thyroid carcinoma (PTC) and positive for TTF-1 but no lymphovascular invasion (Fig 3). 

It was interesting to note that there were no lymphatic structures or cells found within the specimen to deduce that the tumor may have metastatically spread to a parapharyngeal lymph node. 

A multidisciplinary team meeting was held, and the patient was planned for total thyroidectomy followed by radioactive iodine ablation as the primary tumor in the ectopic thyroid had been completely excised. 

However, definitive surgery was delayed by 9 months due to the COVID pandemic, and she returned with palpable right cervical lymph nodes. Repeated USG showed an upgrade of the left thyroid nodule to ACR-TIRADS 4 and a new suspicious right cervical lymphadenopathy with the loss of normal fatty hilum. 

**Fig 4:**
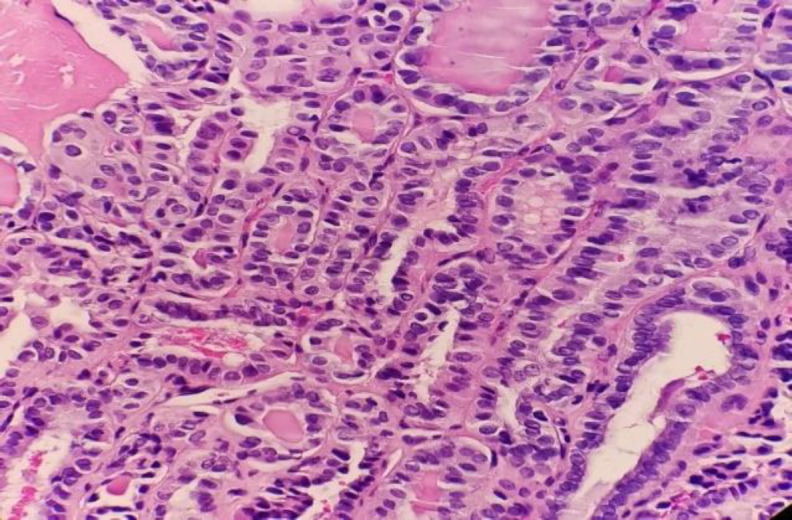
PTC in bilateral thyroid lobes (x40 magnification).

The patient underwent total thyroidectomy with selective neck dissection to the right cervical level II, III, IV, and bilateral level VI central nodes. The thyroidectomy HPE showed small multifocal classical PTC foci involving both lobes with tumor sizes ranging from 0.5 to 1.5 mm, no lymphovascular invasion, and only 1 of 29 lymph nodes with metastasis (Fig. 4). 

The patient subsequently received 150 mCi of radioactive iodine (RAI) ablation, and a whole-body scan did not reveal any distant metastasis. At the time of writing, 1-year post-surgery, she continues to be well, and serum thyroglobulin (TG) shows a reducing trend of 9µg/L from pre-RAI values of 15µg/L, and no structural recurrence has been detected from follow-up imaging thus far. The patient continues to be on TSH suppression therapy and is scheduled for a repeat RAI whole-body scan 6 months post-RAI ablation with further monitoring of the TG trend. 

## Discussion

Development of the thyroid gland in utero begins around weeks 5 to 6 of gestation and starts to secrete thyroid hormones during weeks 10 to 12 (2). The gland derives from the endoderm of the first and second pharyngeal pouches, where it descends along the thyroglossal duct, beginning at the base of the tongue, prior to moving caudally towards the thyroid cartilage at the C5-T1 vertebral level and dividing into the left and right lobes. Although the underlying mechanisms of thyroid dysmorphogenesis are still unclear, several etiologies may be able to explain the different sites at which ectopic thyroid tissue has been found (3). 

The speed at which the thyroid anlages descend should explain the locations within the thyroglossal tract. Over-descent of thyroid tissue along with the cardio-pulmonary trunk into the mediastinum may be due to attachment of the primordial thyroid before caudal migration. Defective migration of lateral thyroid tissue and failure of the thyroid anlage’s median fusion may explain submandibular and lateral neck ectopic thyroid. 

The presence of ectopic thyroid in distant and rare sites may hypothetically be due to aberrant differentiation of uncommitted endodermal cells in those particular organs during early embryogenesis. Genetic factors involving TTF-1, TTF-2, and PAX-8 are thyroid transcription factor genes that modulate thyroid development and thyroid hormone synthesis; thus, mutations in any of these genes may result in dysmorphogenesis (4). 

Standard biochemical workup in a patient suspected of ectopic thyroid includes a thyroid function test, as hypothyroidism has been reported in patients with lingual ectopic thyroid, although most patients would be euthyroid (5,6). USG is commonly used as first-line imaging to delineate the anatomy of the thyroid gland as well as associated structures in the neck. Additional modalities, such as CT or magnetic resonance imaging, may provide more information about the surrounding structures (7). 

Functional imaging techniques, such as thyroid scintigraphy with technetium or radioactive iodine, may help to locate ectopia and investigate the functional status of thyroid tissue. Fine needle aspiration cytology remains a vital tool for assessing any ectopic thyroid tissue. This allows the cytological assessment to identify the presence of thyroid follicular cells and differentiate between benign and malignant lesions (1). As in our patient, it is highly unusual for ectopic thyroid tissue to be present at the posterior pharynx as it is not within the thyroglossal tract. Primary squamous or adenocarcinomas arising from loco-regional sites, such as the oropharynx and upper esophagus, must be excluded. 

Cervical node lymphomas and other soft tissue sarcomas remain viable differentials within the regional anatomy. Differentiating a primary malignant ectopic thyroid carcinoma from a metastatic spread can be especially difficult in areas such as the lateral cervical region and mediastinum, owing to the possibility of lymph node metastases and retrosternal extension, as in our patient, despite the HPE of the resected posterior pharyngeal mass not reporting any evidence of lymphoid tissue (8). However, the importance of this may only be academic, as the principles of management remain unchanged. It is unknown whether the multifocal PTC in both lobes was a result of metastases from the initial tumor in the posterior pharynx or primary tumor growth of previously undetected small microcarcinomas in the gland that USG inaccurately graded. 

Contralateral cervical nodal metastases would result from regional metastatic spread from the multifocal PTC in the gland. 

The management of malignancy in ectopic thyroid tissue is individualized. Complete surgical excision of the ectopic tissue with total thyroidectomy remains the primary modality of treatment in patients with operable tumors, followed by radioactive iodine ablation. 

In cases where cervical nodal disease is present, radical cervical node dissection is advocated (9). Radiotherapy and targeted therapies may be used to palliate patients where tumors are inaccessible or inoperable (10). 

## Conclusion

Ectopic thyroid tissue is uncommon and may be associated with hormonal disorders and malignancy. Most ectopic thyroid tissue arises in the head and neck, despite distant site occurrences that have been documented. 

The principles of management remain those of standard differentiated thyroid cancer protocols: complete surgical resection of the tumor focus, total thyroidectomy, and node dissection of involved lymph nodes, followed by adjuvant radioactive iodine in iodine-sensitive tumors, and a follow-up with routine biochemical and structural assessments of tumor response and recurrence. 
